# The Whys and Hows of Theory—Comparing Cognitive Science and Economics

**DOI:** 10.1007/s42113-024-00213-9

**Published:** 2024-07-29

**Authors:** Patricia Rich

**Affiliations:** Department of Philosophy, University of Bayreuth, Bayreuth, Germany

**Keywords:** Philosophy of science, Economics, Cognitive science, Theory, Methodology

## Abstract

Given the ongoing debates about the replication crisis, theory crisis, and cooperation among disciplines in cognitive science, it is instructive to compare cognitive science with economics. The two fields face common challenges, most importantly in that both study complex, open systems. The strategies for facing these challenges, however, are quite different. Economics was long dominated by theory. Cognitive science takes a multidisciplinary approach, and despite its attendant diversity is dominated by psychology, which itself often neglects theory. I defend economics’ use of theory, characterizing its formal modeling tradition as an effective divide-and-conquer strategy for understanding complex, open systems. I argue that theory and experimentation ideally support one another, making replicability less of an issue. I also discuss the appropriate level(s) of analysis in economics and cognitive science as products of the systems they study. Finally, I compare the two fields’ very different community structures, treating economics as a cautionary tale and advocating pluralism.

## Introduction

The 2022 Lorentz workshop *What Makes a Good Theory? Interdisciplinary Perspectives* centered on both the importance of theory development and practical aspects thereof. This focus is timely, due to ongoing methodological discussions surrounding the replication and theory crises, practical steps that could be taken by individual researchers to address these crises, and potential reforms to scientific institutions to solve these and other problems.

If we are interested in the whys and hows of theory development, then it is instructive to compare cognitive science and economics, since their methodological and cultural differences are so conspicuous. Economics is united by a strong theoretical tradition and a fairly unified modeling paradigm, whereas cognitive science is internally diverse and theory plays a major role in some parts but not in others. It is worth noting that these differences do not seem to be necessary; in fact, one might have thought that economics and cognitive science would be much more similar. As I explain below, the two have a lot in common, including not only overlapping subject matter but also the fact that they study large, complex, open systems. Given this, we can imagine possible worlds in which the two operate in similar ways. We can imagine a more integrated cognitive science with more of a unified theoretical core (compare to Núñez et al. , 2019). Similarly, we can imagine a vastly more interdisciplinary economics in which neuroscientists, anthropologists, statisticians, philosophers, decision theorists, and others collaborate in the common pursuit of understanding the economy and economic behavior. We could even be moving towards one or both of these possibilities. At present, however, economics and cognitive science are very different, and these differences are also closely connected to their asymmetric discussions of the replication crisis, methodological reform, interdisciplinarity, open science, and so forth. Hence, I will take the occasion of the interdisciplinary workshop and corresponding special issue to compare economics and cognitive science in a way that I hope is helpful and informative. In particular, I hope it will help cognitive scientists to imagine and evaluate alternative paths.

A few caveats are in order. First, both fields have obviously been shaped by their history. I don’t want to suggest that this history does not or should not make a difference or that one field can simply implement the strategies of another. At the same time, though, fields continue to change and can definitely learn from one another. Second, some parts of the article focus on psychology specifically. There are a couple of reasons for this. One is that psychology is “dominant” within cognitive science by some measures (Gentner, 2010; Núñez et al., 2019). The other is that the motivating issues of the replication crisis and theory crisis are often seen as issues for psychology especially. Third, I elaborate on economic methodology in more detail, relatively speaking. This is because the audience will be less familiar with economics and because there is more of a unified methodology to describe. Finally, I aim to be brief and to present my own perspective as a philosopher with contact to both economics and cognitive science; it is not possible to discuss any of the topics exhaustively in this setting, and the choices reflect my own viewpoint. Nonetheless, I hope to provide some new food for thought.

This article proceeds as follows:“The Shared Challenge” elaborates on the shared challenge facing economics and cognitive science, specifically that their targets are complex, open systems. “Theory Development Through Formal Modeling,” “Theory and Experiments,” and “Divide-and-Conquer and Levels of Explanation” describe the differences between economics and cognitive science (and especially psychology) with respect to theory development and use and discuss the rationale for and implications of strongly theory-based research. In particular, “Theory Development Through Formal Modeling” explains how theory development in economics is tightly bound up with formal modeling, which in turn constitutes a cumulative, systematic, divide-and-conquer approach to studying complex open systems. “Theory and Experiments” compares economics with psychology in this respect and explains how a foundation of theory (i.e., an established conceptual and ontological landscape as well as formal models) seems to protect economics from a large-scale replication crisis. “Divide-and-Conquer and Levels of Explanation” argues that the nature of complex open systems warrants a divide-and-conquer approach for both economics and cognitive science, but that there may be good, subject-specific reasons why the two use different versions of this approach (with economics focusing on a particular modeling paradigm and cognitive science seeking explanations at different levels, through different means). “Theory as a Community Project” draws attention to a key difference between the economics and cognitive science communities, namely that the economics community is divided between a fairly homogeneous mainstream and a largely ignored heterodox subgroup, whereas cognitive science has always been more pluralist. Pluralism is argued to be important and some suggestions for maintaining it are made, on the basis of the foregoing arguments. “Conclusion” concludes.

## The Shared Challenge

### Subject Matter

Economics and cognitive science face common challenges; despite this, from a methodological viewpoint, they proceed quite differently, which makes it instructive to compare them. This section focuses on the shared challenge, first comparing the fields’ subject matters, followed by the nature of these subject matters as complex and as instantiating open systems.

The most basic commonality between economics and cognitive science is a non-trivial overlap in the subject matters of the two fields, especially in that understanding judgment and decision-making is of central importance to both. As a result, there has been mutual influence between them where this common subject matter is concerned: In cognitive science, formal decision theory—especially the concept of utility maximization—and Bayesianism play a prominent role in some research programs (see, e.g., Gopnik et al. 2004; Oaksford and Chater, 2007; Lieder and Griffiths, 2020).^1^ In economics, there is an influential research program in behavioral economics, which advocates the integration of psychology and a more empirical approach (Camerer, 1999; Angner & Loewensten, 2012; see, e.g., Kahneman et al.  1990; O’Donoghue and Rabin 2000; Ariely et al. 2003; Farber, 2008). A subset of behavioral economists even pursue neuroeconomics, which sees neuroscience as enabling a revolution in decision theory (Camerer et al., 2005; Glimcher & Fehr, 2014).^2^

### Complexity

In my view, the most important parallel between economics and cognitive science is that both study *highly complex, open systems*. This makes them very difficult. Let’s consider complexity first.

*Complexity* is an intuitive concept which can be defined in many ways (see Holt et al. 2011), but Simon (1962) provides a useful characterization for present purposes:

Roughly by a complex system I mean one made up of a large number of parts that interact in a non-simple way. In such systems, the whole is more than the sum of the parts, not in an ultimate metaphysical sense, but in the important pragmatic sense that, given the properties of the parts and the laws of their inter-action, it is not a trivial matter to infer the properties of the whole (Simon, 1962, pg. 468).

Both the economy and cognition are complex in this sense. Economic outcomes are the product of the activities of many individuals, but macro-level economic phenomena have emergent properties and cannot easily be inferred from theories of individual behavior. In fact, even the individuals participating in the economy are themselves complex systems, studied (as noted above) by both economists and cognitive scientists. For the cognitive scientist, the individual is a complex system whose properties cannot be easily inferred from theories of its parts, whether those be biological parts like neurons or particular cognitive processes.

The complexity of both systems has been discussed, with interest in the topic increasing. Simon, who did important early work at the intersection of both disciplines when cognitive science was still quite new, even compares the two to illustrate the issue. In his discussion of complexity, social systems and biological systems both serve as examples (Simon, 1962). More recently, economists—especially those with a heterodox bent—have argued for a more pluralist approach to studying the economy to ensure that this complex system is studied from more angles (see Garnett Jr, 2006, for discussion). This is analogous to calls in cognitive science to pursue all levels of analysis, the idea being that no single level (e.g., the neuronal or “implementation” level or the abstract computational level) will suffice (Bechtel & Shagrir, 2015). Holt et al. (2011) have even heralded the arrival of the “complexity era” in economics. Similarly, Favela (2020) argues for “cognitive science as complexity science.”

### Open Systems

As with complexity, *open systems* (Von Bertalanffy, 1950) can be defined in different ways (see Chick and Dow, 2005). For present purposes, the crucial observation is that there is basically no limit to the causal factors that can be relevant to the systems we are studying. Put differently, for any model of the system that one could develop, that model will be incomplete in that factors or forces from outside of the model will at least sometimes have an important influence on what happens within the system.

Both the economy and cognition are open systems in this sense. Simon again alludes to the difficulties of studying such systems early on, before the open systems terminology established itself in the social sciences:

It is a common experience in experimental psychology, for example, to discover that we are studying sociology—the effects of the past histories of our subjects—when we think we are studying physiology—the effects of properties of the human nervous system. Similarly, business cycle economists are only now becoming aware of the extent to which the parameters of the system they are studying are dependent on the experiences of a population with economic events over the previous generation (Simon, 1980, pg. 33).

So, both the economy and cognition are not only very complex—with more parts than can be simultaneously investigated interacting in ways that are often hard to grasp (as anyone studying either should know well)—but always potentially influenced by factors that one had hoped to be able to ignore. These could be historical factors (in both cases), events outside the natural borders (e.g., national borders for the economist or individual borders for the cognitive scientist) or aspects of the environment or of agents that were not on the radar or consciously ignored for the purposes of inquiry (e.g., the role of emotions in decision-making). It is neither possible to consider everything simultaneously nor to be completely sure that what is left out would not significantly change the picture.^3^ Researchers in both economics and cognitive science must do their best given this difficult state of affairs.

With this common challenge in mind, let’s turn to how theory is (or is not) used to tackle it, the rationale for theory development as a strategy, and the implications that this strategy has. In particular, “Theory Development Through Formal Modeling” characterizes formal modeling (closely related to theory development) as a divide-and-conquer strategy for studying complex open systems. “Theory and Experiments” discusses the connection between economics’ and psychology’s relationships to theory and the replication crisis. “Divide-and-Conquer and Levels of Explanation” discusses the connection between the divide-and-conquer approach and levels of explanation.

## Theory Development Through Formal Modeling

Theory can be developed in many ways. Formal models are one important tool often used for this purpose; they provide a way for theoretical commitments to be clearly laid out and for a theory’s implications to be explored. Modeling results can then also cause theories to be revised, refined, or expanded. Within cognitive science, formal, analytical modeling is an *option*, pursued in some areas (e.g., formal linguistics or mathematical psychology) and not in others (e.g., experimental branches of psychology or the primarily qualitative anthropology^4^). In contrast, providing a formal model is practically *required* in economics. Economics is special among the social sciences in that formal modeling has long been *the* method of research; a “formalist-deductivist” (Lawson, 2006) approach to its target topics (e.g., markets, choice) can even be taken as constitutive of economics (see also Robbins, 1932; Nelson, 1995). Furthermore, economics largely adheres to a shared modeling paradigm in which individual choice is the driver and individuals are assumed to be rational in the sense of making coherent choices, typically in the sense of maximizing some utility function.^5^ Experimentation has become an important part of the mainstream toolkit over the past few decades, but experiments tend to be based on theory or on theory-based model results and often serve further theory development. Robust empirical findings often lead to new variations or expansions of existing models.

Before discussing the benefits and drawbacks of formalization from the present-day perspective, it is useful to consider the historical origins of the status quo. An important reason for economists’ methodological choice, from a historical perspective, is certainly the earlier belief that “real science” requires formalization and precision (see, e.g., Ross, 2018). Physics—which is known for being quite theory-heavy and mathematical—was long seen as the paradigmatic science, and economics tried to emulate physics. Other conceptions of science are possible, however; in fact, psychologists may instead think that “real science” requires performing experiments (Flis, 2019; van Rooij, 2021). Any such extreme and simplistic views about what constitutes science are now generally seen by philosophers of science as outdated. Science is not equal to a mathematical or an empirical methodology, nor to a special combination of the two (see Taylor, 1971, for discussion regarding social science). Instead, different sciences legitimately employ different methodologies, determined by their subject matters and shaped by historical and sociological factors. We have already observed that economics and cognitive science (and psychology) have similar and even strongly overlapping subject matters; hence, their methodological differences are largely due to history and probably say more about the scientists involved than about the phenomena they seek to explain. Indeed, cognitive science *exists* as it is—with a much more diverse methodology than economics—because scientists with different backgrounds recognized that their different perspectives and toolkits all had potential and value for understanding the common target phenomenon of cognition.

With this in mind, let us discuss formal modeling in more detail. Analytical models especially are often criticized for being too simple, too idealized, too abstract, and too different from the real world to tell us much about it. In the literature on the use of models in economics, models are often on the defensive. Because of this—and because I think models have a crucial role to play in the process of developing and deploying theories—it is worth spelling out the positive case for formal modeling as a modern strategy for dealing with the challenge of complex open systems.

Let’s imagine that we are interested in studying the ice cream market—maybe we want to anticipate when, where, and how much ice cream will be sold in the coming years. Because the ice cream market is an open system, we know that there is no limit in principle to the factors that might influence this market. It is inherently impossible, then, to account for all of the factors—and so there is also no point in trying; instead, we will ignore most of these factors according to a pre-established system. From our present point of view, and given our specific interests, some factors may strike us as particularly important (say, temperatures and ingredient costs) while others seem less important (at the extreme end, are consumers attracted to their local parlor because the salesperson has really cool hair, or repelled by the politics of the owner?). The economist can generally use their (schooled) intuition about the target system to place such factors on a spectrum of relevance and interest for present purposes. In fact, when Katzner (2017) describes the steps the economist (ideally) goes through when modeling something, the first step is essentially thinking about the target phenomenon and using judgment to decide what factors seem to be most important for explanatory purposes. In practice, the economist makes the choice to ignore all but the top factor(s), so that through the next steps, they can create a model which is on the one hand tractable and enables results to be derived, but on the other hand captures what one hopes are the most relevant mechanisms for present purposes.

For Katzner, the economist then (again, ideally) follows a process of preliminary model sketching, detailed formalization, empirical evaluation (as possible), and a holistic evaluation of the explanatory value of the model. We can add “repeat” as a final step, to be performed either by the original economist(s) or by others: critically, since we know that we have ignored so many potentially important features of the system, the resulting model is a starting point rather than an end point. Just as importantly, though, we would build *systematically* from the starting point, for example, by identifying the intuitively most relevant factor left out of the initial model, and seeing how to incorporate it. (In the ice cream market example, this might mean building in the trend of avoiding animal products.) Relatedly, economists often perform *robustness analysis* to evaluate the impact of questionable assumptions, checking how the model results might change when different factors are included or different assumptions are made (see Weisberg, 2006; Kuorikoski et al.  2010, for breakdowns of the process of robustness analysis).

Over time, this means that economists study systems of interest by building up a *library* of models that collectively cover more and more of the space of possibilities and accommodate increasing levels of complexity (Veit, 2020). And because this process is used systematically by the whole mainstream economics community, there is a clear sense in which the library of models as a whole can be used to tell a story; the models are not disjointed, but overwhelmingly stand in clear relationships to one another, supplementing and complementing each other. If an economist perceives that an apparently important aspect of the real system has been left out of or inadequately studied by existing models, it is generally clear how to remedy the problem—by expanding or combining elements of existing models to include the new factor, according to established modeling principles.^6^

So, economists deal with the challenge of getting a grip on complex open systems with a highly regimented approach that has them prioritize potential system features, focus, prove, and repeat. While each resulting model is clearly limited, the idea is that understanding can be built up through time and patience.^7^ If models are not tweaked and built upon, then it never becomes clear how robust their conclusions are, what their limitations are, what their descendants can be used for, and so forth. Repeatedly starting over with new models rather than following up on existing ones would be like repeatedly trying on new sneakers but without adjusting the tongue, tying the laces, thinking about your socks, and walking around; maybe the first pair would have been perfect for a marathon, but you would never learn this. For all the methodological discussion warning of the dangers of over-reliance on modeling results and the difficulty of drawing strong inferences about reality from the models, philosophers of science generally acknowledge that there is no magic solution to avoid the dangers and difficulties, and tend to endorse tweaks, supplements, and perspective shifts for the dominant modeling methodology rather than replacing it wholesale (see, e.g., Alexandrova, 2008).

Computational cognitive scientists may appreciate this perspective on economics because it shows that slow, pen-and-paper theoretical work (including formal model building, but not only) bears fruit over the long term and requires patience. Compared to the empirical data generated by an experiment, it may feel like time spent theorizing has not “produced” as much.^8^ No matter how much the new bit of theory or model improves on what came before, some fellow researchers will look at it and see that it leaves out something they find to be important, or that it makes some assumption they don’t trust. The example of economics shows that it can be necessary to take a longer view and to view the new work as one piece of a puzzle, one model in the library, and as a work in progress. Just imagine if game theory hadn’t been pursued because the initial models were primitive; then evolutionary biology as we know it could not exist, for example, nor could many other areas of research. Economics had a head start of several decades in formal modeling. Beyond this, its key formal tools were developed earlier for mathematics and physics.^9^ Cognitive science, in contrast, relies not only on calculus but more importantly on formal tools from computer science, which is itself younger than economics.^10^ Hence, theoretical (and especially formal) work in cognitive science may not look as valuable as it really is; it is important to consider the value that can be created by building on it in the long term. And, of course, to actually try building on it, even if the new models also look too simple, in order to potentially realize that value.

Formal modeling represents a particular divide-and-conquer approach to studying complex phenomena that has persisted despite the limitations of models. This makes sense because creating a formal model makes especially visible that some parts of the system are being ignored, but it’s not as if by failing to model we could somehow take the entirety of a complex, open system into account all at once. Quite the contrary, as Reijula et al. (2023) explain, a divide-and-conquer approach has long been defended by philosophers and cognitive scientists, for example by Simon (1962), Wimsatt (2007), and Bechtel and Richardson (2010); the latter three authors emphasize the *heuristic* value of treating complex systems as decomposable so that they can be studied.

I will return to Simon’s defense of a divide-and-conquer approach in “Divide-and-Conquer and Levels of Explanation”. For now, the point is that formal modeling can be a legitimate strategy for dealing with complex, open systems even though that strategy inherently involves ignoring much of the complexity. The question is whether that ignoring is really targeted and strategic, i.e., whether the formal modeling is done systematically (or “cumulatively” to use Kaznatcheev’s (2022) term). Again, this is partly because using a series or collection of models provides a kind of antidote to the simple and idealized nature of each individual model (see, e.g., Jhun, 2023, for discussion). Explanations of real-world phenomena must draw on such an expanded pool of resources; a robust and useful theory can neither be based on isolated models nor on isolated experiments. In fact, Newell (1973) already bemoaned that psychology wasn’t proceeding cumulatively, saying of contemporary research:

What I wanted was for these excellent pieces of the experimental mosiac [sic] to add up to the psychology that we all wished to foresee. They didn’t, not because of any lack of excellence locally, but because most of them seemed part of a pattern of psychological activity that didn’t seem able to cumulate.

Psychology has become more quantitative, as Newell also encouraged, but not in such a way that it produces long chains of cumulative theory, as economics does. Instead, many cognitive scientists lament that psychology’s increased formalization has been largely restricted to empirical inference, i.e., focusing on links between hypotheses and data formalized in terms of things like statistical significance levels and other statistical tests and properties (see, e.g., Oberauer and Lewandowsky, 2019, pg. 1597). Let us turn now to the different relationships between theory and empirical work in economics and psychology, and the consequences of these relationships.

## Theory and Experiments

Given that theoretical work is well established in economics, it is unsurprising that empirical work is part of a loop with theoretical work. Experimentalists can rely, at minimum, on the ontology established by economic theory, and they will have a good idea of how theoreticians view the world. Theoreticians, in turn, are likely to take note of empirical results (or observations more generally) indicating interesting phenomena or factors that have been left out of existing theory. While abstract theory was long dominant, the field has moved to a state of greater balance and cooperation. In psychology, the balance is a different one. Flis (2019) explains that the focus in psychology is on generating and interpreting data. Discussion of how to generate hypotheses to test in the first place has been neglected.

I don’t want to make a general prescriptive argument for a specific relationship between theory and experiments.^11^ Instead, I think it is useful to consider the disciplinary differences in this regard from two perspectives: First, how do the differences relate to the replication crisis? Second, what are the potential downsides to relying on theory when doing empirical work, and how can they be minimized?

The replication crisis needs little introduction. It is also understood to have many causes. Some of these—like the file drawer effect or even outright fraud—presumably have little to do with discipline-specific norms or preferred methodology. Some have interpreted the replication crisis as reflecting a theory crisis, however (see, e.g., Muthukrishna and Henrich, 2019; Oberauer and Lewandowsky, 2019; van Rooij, 2019; Lavelle, 2022). Smaldino (2022) shows, for example, that without strong prior theoretical support for the hypotheses being tested, false hypotheses are more likely to be supported by the data just by chance, leading to low replicability (see also Bird, 2021). In light of this argument, economics’ strong theoretical underpinnings should provide protection from a replication crisis, and some proposed responses reflect the role to be played by theory. Along these lines, Devezer and Buzbas (2023) advocate a process of iteratively building and refining models to improve generalizability in psychology. Guest and Martin (2021) advocate computational modeling, partly to improve replicability and to make the implications of a failed replication for theory easier to determine. Similarly, van Rooij and Baggio (2021) advocate building and evaluating computational-level theories of cognitive capacities (rather than effects) before empirically testing those theories.

A useful question at this point is whether economics has in fact been shielded from the replication crisis. We wouldn’t expect it to be immune, since, for example, the file drawer effect is clearly a problem in economics as elsewhere. Instead, we would expect replicability to be higher but not perfect. In fact, there seems to have been little discussion of a general replication crisis in economics, with the problem instead strongly associated with psychology and medicine. However, there have been a few papers about the replication crisis pertaining to some subfields of economics, such as finance and experimental economics. Bardsley (2018) directly discusses comparative replicability between psychology and economics. He compares three replication studies of work in psychology and one in economics. Indeed, replicability in the economics study was superficially much higher than in two of the three psychology studies (though Bardsley points out that the results are influenced by methodological choices, which also differed across studies). In general, Bardsley complains that independent, direct replication attempts in economics are very rare.^12^ He agrees, though, that economics is well set up for direct replication of experiments. Nonetheless, he sees a problem for replicability in economics. Specifically, while economic models may generate precise predictions about what should happen in stylized experimental set-ups—which can then be tested—this also means that most experiments stick very close to the original model.^13^*Conceptual* replication—which aims to support a hypothesis through a different, perhaps more natural experimental set-up—is neglected. In terms of the real-world performance of economic theory, however, such conceptual replications would be more informative. This is less of a problem in psychology, where there is no such barrier to performing more natural kinds of experiments.

An overly strict relationship between theory and experiments may have other downsides. For example, the more experiments are determined by theory, the less room may be left for creativity and even luck. Yet these play an important role in science, perhaps especially when it comes to truly new insights. In fact, creativity seems to be valued more in psychology than in economics; behavioral economics is an interesting case in this respect. Behavioral economists see themselves as economists, but they fall outside of the orthodox, and much of their work fits just as well in cognitive science. Setting aside some controversial projects associated with behavioral economists (e.g., the “nudge” program and neuroeconomics), their work seems to be beloved far beyond academic economics or cognitive science. It seems that behavioral economics represents a highly productive mixture of having theoretical foundations and permitting creativity. The theoretical basis comes from the shared education in economics and the use of its ontology and concepts. Creativity, in particular an eagerness to speculate about the psychological origins of people’s choices, is apparently a cultural value in the community. Hence, behavioral economics research can be readily understood in relation to orthodox research and also integrated into it, while at the same time, it has been able to break new ground.

This impression fits well with recent discussions about the need to avoid making research too objective or proceduralized (see, e.g., Field and Derksen, 2021; Rich et al. 2021). Too much regulation in response to the replication crisis would bring its own problems, such as stifled creativity and reduced innovation,^14^ at a time when some are already worried about science becoming too conservative and not disruptive enough (see, e.g., Stanford, 2019; Park et al. 2023). Theory can be promoted and its value can be recognized without it becoming a straight jacket.

## Divide-and-Conquer and Levels of Explanation

Let’s return now to the idea of understanding a complex system through a divide-and-conquer approach. The idea was brought in earlier as a way to support the use of formal models that (necessarily) ignore many parts of the target systems. In this section, the focus is on a special type of ignoring or dividing, specifically dividing the target phenomenon into distinct *levels* and then either studying those levels independently or, perhaps, ignoring some levels.

In both economics and cognitive science, there are thorny and even overlapping discussions of the right level of analysis or explanation—for example, must the system be explained in terms of its most basic parts? Would this even be possible? In cognitive science, the discussion centers on Marr’s levels of analysis (the computational, algorithmic, and implementation levels) (Marr, 1982). In economics, except for the aforementioned neuroeconomics, the salient levels are those of individual decision-makers and macroeconomic phenomena, and the key question is whether or not explanations must be grounded in the individual level (Lucas Jr, 1976; Hoover, 2008; List & Spiekermann, 2013; Ruiz & Schulz, 2023).

Some may have the feeling that levels of explanation have been discussed to death, both in cognitive science and in (at least the philosophy of) economics. I won’t attempt to re-hash the standard arguments. Instead, I think it is instructive to consider the levels debate from the perspective of Simon’s (1962) discussion of complex systems and how we can study them, mentioned briefly above. In doing so, I want to make two points. First, the nature of the specific system under study can make particular units or levels of analysis especially salient—and Simon’s perspective provides new arguments for focuses already advocated in the two fields on other grounds. Second, at the same time, we can use Simon’s perspective to provide a new argument for the legitimacy of studying systems at multiple levels simultaneously, a view which is fairly standard in cognitive science but not in economics.

Simon (1962) argues that complex systems which are hierarchically organized could evolve more easily, giving us some reason to think that the complex systems we are trying to study have hierarchical structure. By hierarchical structure, he means that the complex system is made up of nested subsystems. Furthermore, the systems will be “nearly decomposable” in the sense that there is much more important interaction within a subsystem than between subsystems; the impact of one subsystem on another can be largely understood as an aggregate impact (the subsystem as a whole has an impact) rather than in terms of the individual impacts of the parts. Especially in light of the challenge of open systems described above—that there is no limit to the ‘external’ factors that could have an important impact on the system under study—it is clear why a hierarchical and nearly decomposable system would be much easier to study than an arbitrary complex system. If the system has such a structure, then the subsystems can be fruitfully studied individually, even though they are connected.

Simon (1962) discusses near decomposability of the economic system. The price for any good, he says, tends not to depend strongly on the prices of many other goods individually, but instead on the prices for a few key individual goods as well as the general price levels (an aggregate). In contrast, the consumption subsystem presents a violation of near decomposability, because consumption interacts with the other subsystems in a deeper way. From a researcher’s perspective, this would mean that it is problematic to try to study other subsystems while setting aside consumption or oversimplifying it. In fact, economists don’t neglect the consumption subsystem; they do the opposite. Arguably, they have made consumption the very center of economic research. For example, Robbins (1932) defines economics as the study of choice under scarcity, assuming that agents have insatiable wants but limited means. That is, economics is the study of consumption behavior.

While there is a small slide from consumption to consumption behavior specifically, the emphasis on consumption means that the driver of the system (according to the dominant theoretical approach) is agents’ choices. This is taken to the extreme in the methodological tenet known as “microfoundations”; economic theories, models, and explanations must have a choice-theoretic foundation. Hence, we could see Simon’s observations about the economic system as providing an alternative justification for economists’ focus on the individual level.

Though individual cognitive scientists may prefer to research at some specific level (e.g., a neuroscientist might emphasize the implementational level,^15^ while a computational cognitive scientist focuses on the computational level), there is no analogue of the rigid microfoundations tenet. It is easy to think that this difference is mainly just a further consequence of economics’ relative methodological homogeneity, in general, and cognitive science’s interdisciplinary nature. Perhaps, though, it is also a product of a real difference between the systems under study. Perhaps cognition has no counterpart to consumption, in the sense that there is no special subsystem of cognition that deeply influences all other subsystems, precluding their (successful) independent study? In fact, accounts of cognition have been proposed which reflect the idea that cognition is nearly decomposable in a stronger way. Minsky’s (1988) “society of mind” approach is premised on the idea that the mind is composed of many subsystems which have specific tasks; the interaction of these subsystems (rather than of their parts) enables intelligent human behavior. The modularity of mind thesis (Fodor, 1983) is similar in that to the extent it is correct, the mind’s modules can be studied individually. On the other hand, in the case of cognition (and in contrast with the economy), whatever subsystems or modules exist are coordinated and unified, jointly enabling the goal directed and coherent behavior of an individual person. In line with Fodor’s reasoning that the mind’s central processing cannot be modular, whatever coordinates and unifies the mind’s subsystems might be a candidate for cognition’s analog of consumption, deserving a central place in cognitive science. Whereas in economics the permeation of consumption provided an argument for a focus on the individual level, in cognitive science a permeation of central processing would (to me) seem to provide an argument for computational level modeling—what the central processing *does* would seem to be an essential question and a prerequisite for understanding much of the rest. Indeed, Minsky’s focus is at this level, with an attempt to describe abstractly what the (parts of the) mind are doing.

Of course, none of this is to suggest that economists should focus exclusively on the individual level (as opposed to, e.g., the brain, genes, groups, economic aggregates, or structures), nor that cognitive scientists should only do computational modeling. Instead, Simon’s picture of subsystems highlights that there are multiple levels, and less trivially, that it is (a priori) methodologically legitimate to study those different levels. Near decomposability would mean that genuine higher-level (sub)systems can accurately be represented as influencing one another, without representing lower-level interactions between their parts. It remains an empirical question, though, which subsystems there are and how they interact, the extent to which the systems are hierarchical and nearly decomposable, and how different the economy and cognition turn out to be in terms of abstract structure.

## Theory as a Community Project

Before concluding, I would like to emphasize that developing and using theory is a community project and draw some lessons from economics regarding how to maintain a strong community in cognitive science. With respect to community, cognitive science currently has a major advantage over economics, namely that it fosters interaction and engagement between researchers with diverse backgrounds and methods; in fact, we can understand this as central to cognitive science’s response to the challenge of studying complex open systems. As I suggested in the introduction, economics could be just as interdisciplinary as cognitive science; its natural subject matter (the economy and economic behavior) could certainly be explained from multiple distinct perspectives and with the help of various tools. Instead, economics stands out in that there is a sharp division between the orthodox (and somewhat broader mainstream) community and the heterodox community. Heterodox economists employ a much wider range of methods and hence are methodologically heterogeneous, and they embrace interdisciplinarity. Dow (2008) explains that this methodological difference goes along with an asymmetry in perspective and awareness: Heterodox economists see both groups *as* economists, while orthodox economists often define their discipline such that heterodox approaches do not count. Hence, orthodox economists are typically completely unaware of what happens within the heterodox community, including the development of alternative methodologies (e.g., advances in qualitative research methodology; see also Nelson (1995)).

What impact does this have on economic research? On the one hand, it is possible to see the discipline and cohesiveness induced by the presence of a rigid orthodoxy as responsible for the field’s systematic, theory-driven, divide-and-conquer strategy to studying complex open systems, as described above—recall Kaznatcheev’s (2022) characterization of the degree to which a discipline is theoretical as the degree to which work builds on previous work. The orthodox perspective renders it *necessary* for most economists to build on previous work in particular ways, working within the standard modeling paradigm. On the other hand, this strategy also carries particular costs. For one, the use of a formalist-analytic modeling methodology means that explanations not attainable in this way will remain out of reach.^16^ There surely exist aspects of the economy which could only be understood through other, different, methods. Dow’s (2008) point about orthodox economists being broadly unaware of heterodox developments also reflects an important cost to the current community structure. Not everyone can devote significant research time to following developments that don’t seem directly relevant to their own projects. However, as Dow explains, when the orthodox community faces a hard problem (as when theory cannot explain something that we observe), researchers may be unaware of resources from the heterodox community that could help to solve the problem, which can slow progress or even prevent it altogether. The division in the community according to methodology can also be seen as artificial and inefficient; if, for example, scientific exchange depended on the aspect of the system being studied (e.g., employment decisions or the impact of bank regulations) rather than (primarily) on common methods, one can well imagine that economics in general would come to understand those aspects more quickly and more fully. Hence, as mainstream economics grows and naturally evolves, for example, to cover new topics and to incorporate empirical evidence more strongly, there are good reasons for the community to become more pluralistic (as advocated by many heterodox economists; see, e.g., Dow (2008); Garnett Jr (2006)).

Cognitive science, in contrast, should take care not to move too far in the other direction, and take steps to avoid the kinds of power imbalances and asymmetric awareness that have held economics back. As Dale (2008) notes, the current plurality of cognitive science does not imply a pluralist *attitude* according to which there must always be many approaches and none is going to win out. He provides a systematic integration of arguments for pluralism in cognitive science; the example of economics provides a further argument. That field has tried extreme non-pluralism, and it has become clear that it causes serious problems, and that the future will be more pluralistic rather than less; this story should serve as a cautionary tale for cognitive science.

There are open questions regarding whether cognitive science should be multidisciplinary or interdisciplinary (cf. Núñez et al. 2019; Cooper, 2019) and which disciplines should have what sized role (see, e.g., Beller et al. 2012; Cooper, 2019). The arguments presented in this paper do not directly answer these questions, but they do point to some crucial considerations.

First, a powerful strategy is needed to tackle complex open systems, and such a strategy will probably have a divide-and-conquer character. If cognitive science is pursuing a strategy that fundamentally relies on diversity (for example, of levels of explanation), then lowering its diversity would undermine its strategy. For the sake of preserving diversity, it is important that students and junior researchers are exposed to a range of possibilities for studying cognition and can make a free choice (cf. Garnett Jr, 2006). This means that the longer-term costs of fragmentation should be considered when sacrificing breadth for depth in the education of future cognitive scientists. Furthermore, it is critical that respectful lines of communication between member disciplines not only stay open, but permit bi-directional communication (this is another reason why the education of future cognitive scientists is crucial). Special attention may be required here, for example, to ensure that qualitative research is not presumed to be unscientific; this happens easily if quantitative researchers are largely ignorant of qualitative methods and communication is limited (cf. Madill and Gough, 2008; Nelson, 1995).

Second, theory development is beneficial (recall the discussion of the replication crisis), but productive theory development requires time and resources because its benefits are cumulative. Hence, insofar as there is competition within cognitive science, it is important to protect theoretical work. It is important, for example, that any numerical advantage that, say, psychology would have by virtue of being over-represented not translate into (and then be perpetuated by) a structural advantage, for example, when criteria for publication or research funding are set. Such a structural advantage may arise, for example, if grant proposals are evaluated partly according to whether the methodology and anticipated outputs can be described in detail in advance; theoreticians often cannot do this before carrying out the research in question. Similarly, it may be unwise to put too much emphasis on novelty or riskiness in research, insofar as further developing existing theory is not seen as novel or risky.

## Conclusion

This special issue and the Lorentz workshop on which it is based aim to make progress on the question of what makes a good theory by taking an interdisciplinary perspective. In this paper, I have held up economics as an example of a discipline in which theory is especially strong. Importantly, economics is not theoretically strong because its target system is easy to figure out; instead, theory is such an important tool for economics because its target system is otherwise so hard to grasp. The source of this difficulty—that the target is a complex, open system—is shared with cognitive science. Building up understanding of such systems warrants a systematic divide-and-conquer approach in which theorizing, formal modeling, and empirical work all play a role. Exactly how theory should divide up the target system must be determined with the help of knowledge of the target system, though, meaning that economics and cognitive science may differ with respect to questions like the appropriate level of theoretical unity.

Economists and cognitive scientists can learn a lot from one another, especially when the goal is to shape the future of the field. For cognitive scientists who want to promote theory development, the example of theory in economics is especially useful: It draws attention to the large existing literature examining and defending the use of formal models. It shows that patience is required and the value of theoretical work is not always immediately and easily recognizable. It illustrates how cooperation between theory and empirical work increases trust in both. Lastly, it makes clear that there is a trade-off between homogeneity (in the form of a shared modeling paradigm) which allows the steady pursuit of common projects, and pluralism as required by the study of complex, open systems.

## Footnotes

1 It is not a coincidence that the parts of cognitive science which are closely connected to economics are especially theory-driven.

2 While these relatively new research programs have spurred a significant increase in the interest and incorporation of empirical findings into economics, the greatest impact has come from those findings which can be integrated into the existing theoretical approach. Hence, neuroeconomics has had hardly any impact on mainstream or orthodox practices, whereas behavioral economics has led to new forms of utility functions being used, for example, to reflect empirically observed non-standard weightings of probabilities. See Gul and Pesendorfer (2008) for a discussion of why the impact of psychological and neuroscientific research is limited in this way.

3 See for example the literature on learning from idealized models, e.g., Alexandrova (2008); Cartwright (2009).

4 See Beller et al. (2012) for discussion of the role of anthropology in cognitive science.

5 That is, economists largely adhere to *methodological individualism* (Van den Bergh & Gowdy, 2003; Hoover, 2008; List & Spiekermann, 2013; Ruiz & Schulz, 2023) and *methodological rationalism* (Weber, 1947; Gul & Pesendorfer, 2008).

6 In his keynote at the Lorentz workshop, Kaznatcheev (2022) argues that we can see how theoretical a field is by looking at the structure of community engagement with others’ work; more theoretical fields are more cumulative, meaning that they have more long chains of substantial, direct engagement with prior work. This seems to match economics perfectly, although the hypothesis has (to my knowledge) yet to be tested empirically.

7 Note that I’m not claiming that models or theories are getting closer and closer to some objective truth. The claim, instead, is that descriptively, economists aim to provide better models and improved understanding, at least from the perspective of other economists. See for example McCloskey’s analysis of economics from the perspective of rhetoric (McCloskey, 1998).

8 This impression, and especially the perception that funding bodies, tenure committees, etc. may have this impression, was communicated by other participants during the Lorentz workshop from which this special issue arose.

9 See Katzner (2017, Ch. 4) for an argument that economics is mathematical essentially because people maximize utility and the mathematics of function maximization therefore fits perfectly.

10 Thanks to an anonymous reviewer for pointing this out.

11 It is also important to clarify that I am not advocating (or even discussing) a specific, rigid formula whereby theories must be used to derive predictions and then empirical evidence is used to confirm or falsify the theory. The idea is rightly seen as old-fashioned. For a more modern example of how theory can relate to experimentation, consider for example mega-studies as discussed by Duckworth and Milkman (2022). In mega-studies, theories (typically, it seems) inform the treatments to be tested, and so theory is a valuable resource to be used to generate ideas. The point of the experiments is not to test the theories, though, but rather to assess policy options.

12 There were calls for more replication in economics in the 1980’s (Kane, 1984; Dewald et al., 1986) and this may have contributed to better data availability and transparency, but it does not seem to have made actual replication a standard occurrence (see, e.g., Ankel-Peters et al. 2023).

13 See also Sitzia and Sugden (2011).

14 Though see Frankenhuis and Nettle (2018) for a critical discussion of this viewpoint.

15 Though of course they need not do so; see Churchland and Sejnowski (1988); Piccinini and Shagrir (2014) for discussion.

16 Prominent heterodox critic Lawson even argues that the nature of economics’ subject matter—the very fact that it studies complex, open systems—makes the orthodox methodology inappropriate (Lawson, 2006). Indeed, Lawson argues on this basis that orthodox economists don’t see their target systems in this way, but since it seems so obvious that the economy is not a closed system, I find it more charitable to interpret them as thinking that their divide-and-conquer strategy is reasonable for open systems.

## References

[CR1] Alexandrova, A. (2008). Making models count. *Philosophy of Science,**75*(3), 383–404.

[CR2] Angner, E., & Loewensten, G. F. (2012). Behavioral economics. In: U. Mäki (Ed.), *Handbook of the philosophy of science: Philosophy of economics* (pp. 641–690). Amsterdam: Elsevier.

[CR3] Ankel-Peters, J., Fiala, N., & Neubauer, F. (2023). Do economists replicate? *Journal of Economic Behavior & Organization,**212*, 219–232.

[CR4] Ariely, D., Loewenstein, G., & Prelec, D. (2003). “Coherent arbitrariness”: Stable demand curves without stable preferences. *The Quarterly Journal of Economics,**118*(1), 73–106.

[CR5] Bardsley, N. (2018). What lessons does the “replication crisis” in psychology hold for experimental economics? In: *The Cambridge handbook of psychology and economic behaviour, 2nd ed.*, page 42. Cambridge University Press.

[CR6] Bechtel, W., & Richardson, R. C. (2010). *Discovering Complexity: Decomposition and Localization as Strategies in Scientific Research*. MIT Press.

[CR7] Bechtel, W., & Shagrir, O. (2015). The non-redundant contributions of Marr’s three levels of analysis for explaining information-processing mechanisms. *Topics in Cognitive Science,**7*(2), 312–322. 25900887 10.1111/tops.12141

[CR8] Beller, S., Bender, A., & Medin, D. L. (2012). Should anthropology be part of cognitive science? *Topics in Cognitive Science,**4*(3), 342–353. 22685087 10.1111/j.1756-8765.2012.01196.x

[CR9] Bird, A. (2021). Understanding the replication crisis as a base rate fallacy. *The British Journal for the Philosophy of Science*.

[CR10] Camerer, C. (1999). Behavioral economics: Reunifying psychology and economics. *Proceedings of the National Academy of Sciences,**96*(19), 10575–10577. 10.1073/pnas.96.19.10575 PMC33745 10485865

[CR11] Camerer, C., Loewenstein, G., & Prelec, D. (2005). Neuroeconomics: How neuroscience can inform economics. *Journal of economic Literature,**43*(1), 9–64.

[CR12] Cartwright, N. (2009). If no capacities then no credible worlds. but can models reveal capacities? *Erkenntnis, 70*(1), 45–58.

[CR13] Chick, V., & Dow, S. (2005). The meaning of open systems. *Journal of Economic Methodology,**12*(3), 363–381.

[CR14] Churchland, P. S., & Sejnowski, T. J. (1988). Perspectives on cognitive neuroscience. *Science,**242*(4879), 741–745. 3055294 10.1126/science.3055294

[CR15] Cooper, R. P. (2019). Multidisciplinary flux and multiple research traditions within cognitive science. *Topics in Cognitive Science,**11*(4), 869–879. 31564063 10.1111/tops.12460

[CR16] Dale, R. (2008). The possibility of a pluralist cognitive science. *Journal of Experimental and Theoretical Artificial Intelligence,**20*(3), 155–179.

[CR17] Devezer, B., & Buzbas, E. O. (2023). Rigorous exploration in a model-centric science via epistemic iteration. MetaArXiv. 10.1037/mac0000121 PMC10888142 38405689

[CR18] Dewald, W. G., Thursby, J. G., & Anderson, R. G. (1986). Replication in empirical economics: The journal of money, credit and banking project. *The American Economic Review*, pages 587–603.

[CR19] Dow, S. C. (2008). Plurality in orthodox and heterodox economics. *Journal of Philosophical Economics,**1*(2), 73–96.

[CR20] Duckworth, A. L., & Milkman, K. L. (2022). A guide to megastudies. *PNAS Nexus, 1*(5), pgac214. 10.1093/pnasnexus/pgac214 PMC9802435 36712333

[CR21] Farber, H. S. (2008). Reference-dependent preferences and labor supply: The case of New York City taxi drivers. *American Economic Review,**98*(3), 1069–1082.

[CR22] Favela, L. H. (2020). Cognitive science as complexity science. *Wiley Interdisciplinary Reviews: Cognitive Science,**11*(4), e1525. 32043728 10.1002/wcs.1525

[CR23] Field, S. M., & Derksen, M. (2021). Experimenter as automaton; experimenter as human: Exploring the position of the researcher in scientific research. *European Journal for Philosophy of Science,**11*(1), 11.

[CR24] Flis, I. (2019). Psychologists psychologizing scientific psychology: An epistemological reading of the replication crisis. *Theory & Psychology,**29*(2), 158–181.

[CR25] Fodor, J. A. (1983). *The modularity of mind*. MIT press.

[CR26] Frankenhuis, W. E., & Nettle, D. (2018). Open science is liberating and can foster creativity. *Perspectives on Psychological Science,**13*(4), 439–447. 29961408 10.1177/1745691618767878 PMC6041740

[CR27] Garnett, R. F., Jr. (2006). Paradigms and pluralism in heterodox economics. *Review of Political Economy,**18*(4), 521–546.

[CR28] Gentner, D. (2010). Psychology in cognitive science: 1978–2038. *Topics in Cognitive Science,**2*(3), 328–344. 25163863 10.1111/j.1756-8765.2010.01103.x

[CR29] Glimcher, P. W., & Fehr, E., editors (2014). *Neuroeconomics: Decision Making and the Brain*. Elsevier, London, 2 edition.

[CR30] Gopnik, A., Glymour, C., Sobel, D. M., Schulz, L. E., Kushnir, T., & Danks, D. (2004). A theory of causal learning in children: Causal maps and Bayes nets. *Psychological Review,**111*(1), 3. 14756583 10.1037/0033-295X.111.1.3

[CR31] Guest, O., & Martin, A. E. (2021). How computational modeling can force theory building in psychological science. *Perspectives on Psychological Science,**16*(4), 789–802. PMID: 33482070. 33482070 10.1177/1745691620970585

[CR32] Gul, F., & Pesendorfer, W. (2008). The case for mindless economics. In A. Caplin & A. Schotter (Eds.), *The foundations of positive and normative economics: a handbook* (pp. 3–42). New York: Oxford University Press.

[CR33] Holt, R. P., Rosser, J. B., Jr., & Colander, D. (2011). The complexity era in economics. *Review of Political Economy,**23*(3), 357–369.

[CR34] Hoover, K. D. (2008). Does macroeconomics need microfoundations? In: Hausman, D. M., editor, *The philosophy of economics: an anthology*. Cambridge University Press, Cambridge, 3 edition.

[CR35] Jhun, J. S. (2023). Multi-model reasoning in economics: The case of compass. *Philosophy of Science,**90*(4), 836–854.

[CR36] Kahneman, D., Knetsch, J. L., & Thaler, R. H. (1990). Experimental tests of the endowment effect and the Coase theorem. *Journal of Political Economy,**98*(6), 1325–1348.

[CR37] Kane, E. J. (1984). Why journal editors should encourage the replication of applied econometric research. *Quarterly Journal of Business and Economics*, pages 3–8.

[CR38] Katzner, D. W. (2017). *Models, mathematics, and methodology in economic explanation*. Cambridge University Press.

[CR39] Kaznatcheev, A. (2022). Community makes good mathematical theory. Keynote at Lorentz Workshop: What makes a good theory? https://www.youtube.com/watch?v=OPVgcT1HS8E.

[CR40] Kuorikoski, J., Lehtinen, A., & Marchionni, C. (2010). Economic modelling as robustness analysis. *The British Journal for the Philosophy of Science*.

[CR41] Lavelle, J. S. (2022). When a crisis becomes an opportunity: The role of replications in making better theories. *The British Journal for the Philosophy of Science,**73*(4), 965–986.

[CR42] Lawson, T. (2006). The nature of heterodox economics. *Cambridge Journal of Economics,**30*(4), 483–505.

[CR43] Lieder, F., & Griffiths, T. L. (2020). Resource-rational analysis: Understanding human cognition as the optimal use of limited computational resources. *Behavioral and Brain Sciences,**43*, e1. 10.1017/S0140525X1900061X 30714890

[CR44] List, C., & Spiekermann, K. (2013). Methodological individualism and holism in political science: A reconciliation. *American Political Science Review,**107*(4), 629–643.

[CR45] Lucas Jr, R. E. (1976). Econometric policy evaluation: A critique. In *Carnegie-Rochester conference series on public policy*, volume 1, pages 19–46. North-Holland.

[CR46] Madill, A., & Gough, B. (2008). Qualitative research and its place in psychological science. *Psychological Methods,**13*(3), 254–271. 18778154 10.1037/a0013220

[CR47] Marr, D. (1982). *Vision: A Computational Investigation into the Human Representation and Processing of Visual Information*. Freeman, San Francisco, CA: W.H.

[CR48] McCloskey, D. N. (1998). *The Rhetoric of Economics* (2nd ed.). Madison: University of Wisconsin Press.

[CR49] Minsky, M. (1988). *Society of Mind*. Simon and Schuster.

[CR50] Muthukrishna, M., & Henrich, J. (2019). A problem in theory. *Nature Human Behaviour,**3*(3), 221–229. 30953018 10.1038/s41562-018-0522-1

[CR51] Nelson, J. A. (1995). Feminism and economics. *Journal of Economic Perspectives,**9*(2), 131–148.

[CR52] Newell, A. (1973). You can’t play 20 questions with nature and win: Projective comments on the papers of this symposium. In: W. Chase (Ed.), *Visual information processing* (pp. 283–308). New York: Academic Press.

[CR53] Núñez, R., Allen, M., Gao, R., Miller Rigoli, C., Relaford-Doyle, J., & Semenuks, A. (2019). What happened to cognitive science? *Nature Human Behaviour,**3*(8), 782–791. 31182794 10.1038/s41562-019-0626-2

[CR54] Oaksford, M., & Chater, N. (2007). *Bayesian rationality: The probabilistic approach to human reasoning*. Oxford University Press. 10.1017/S0140525X09000284 19210833

[CR55] Oberauer, K., & Lewandowsky, S. (2019). Addressing the theory crisis in psychology. *Psychonomic Bulletin & Review,**26*, 1596–1618. 31515732 10.3758/s13423-019-01645-2

[CR56] O’Donoghue, T., & Rabin, M. (2000). The economics of immediate gratification. *Journal of Behavioral Decision Making,**13*(2), 233–250.

[CR57] Park, M., Leahey, E., & Funk, R. J. (2023). Papers and patents are becoming less disruptive over time. *Nature,**613*(7942), 138–144. 36600070 10.1038/s41586-022-05543-x

[CR58] Piccinini, G., & Shagrir, O. (2014). Foundations of computational neuroscience. *Current Opinion in Neurobiology,**25*, 25–30. 24709597 10.1016/j.conb.2013.10.005

[CR59] Reijula, S., Kuorikoski, J., & MacLeod, M. (2023). The division of cognitive labor and the structure of interdisciplinary problems. *Synthese,**201*(6), 214.

[CR60] Rich, P., de Haan, R., Wareham, T., & van Rooij, I. (2021). How hard is cognitive science? In: *Proceedings of the annual meeting of the cognitive science society*, vol 43.

[CR61] Robbins, L. (2008 (1932)). The nature and significance of economic science. In Hausman, D. M., editor, *The philosophy of economics: an anthology*, pages 73–99. Cambridge University Press, Madison, 3 edition.

[CR62] Ross, D. (2018). Economics and allegations of scientism. In M. Boudry & M. Pigliucci (Eds.), *Science unlimited?: the challenges of scientism. *University of Chicago Press.

[CR63] Ruiz, N., & Schulz, A. W. (2023). Micro-foundations and methodology: A complexity-based reconceptualization of the debate. *The British Journal for the Philosophy of Science,**74*(2), 000–000.

[CR64] Simon, H. A. (1962). The architecture of complexity. *Proceedings of the American Philosophical Society,**106*(6), 467–482.

[CR65] Simon, H. A. (1980). Cognitive science: The newest science of the artificial. *Cognitive Science,**4*(1), 33–46.

[CR66] Sitzia, S., & Sugden, R. (2011). Implementing theoretical models in the laboratory, and what this can and cannot achieve. *Journal of Economic Methodology,**18*(4), 323–343.

[CR67] Smaldino, P. E. (2022). Five models of science, illustrating how selection shapes methods. In G. Ramsey & A. De Block (Eds.), *The dynamics of science: computational frontiers in history and philosophy of science* (pp. 19–39). University of Pittsburgh Press.

[CR68] Stanford, P. K. (2019). Unconceived alternatives and conservatism in science: The impact of professionalization, peer-review, and big science. *Synthese,**196*, 3915–3932.

[CR69] Taylor, C. (1971). Interpretation and the sciences of man. *The Review of Metaphysics*, pages 3–51.

[CR70] Van den Bergh, J. C., & Gowdy, J. M. (2003). The microfoundations of macroeconomics: An evolutionary perspective. *Cambridge Journal of Economics,**27*(1), 65–84.

[CR71] van Rooij, I. (2019). Psychological science needs theory development before preregistration.

[CR72] van Rooij, I. (2021). Tools for theory: Cbs open science.

[CR73] van Rooij, I., & Baggio, G. (2021). Theory before the test: How to build high-verisimilitude explanatory theories in psychological science. *Perspectives on Psychological Science,**16*(4), 682–697. 33404356 10.1177/1745691620970604 PMC8273840

[CR74] Veit, W. (2020). Model pluralism. *Philosophy of the Social Sciences,**50*(2), 91–114.

[CR75] Von Bertalanffy, L. (1950). An outline of general system theory. *British Journal for the Philosophy of Science,**1*(2), 134–165.

[CR76] Weber, M. (1947). *The Theory of Economic and Social Organization*. New York: Oxford University Press.

[CR77] Weisberg, M. (2006). Robustness analysis. *Philosophy of Science,**73*(5), 730–742.

[CR78] Wimsatt, W. C. (2007). *Re-engineering philosophy for limited beings: Piecewise approximations to reality*. Harvard University Press.

